# Supervised machine learning for automatic classification of in vivo scald and contact burn injuries using the terahertz Portable Handheld Spectral Reflection (PHASR) Scanner

**DOI:** 10.1038/s41598-022-08940-4

**Published:** 2022-03-24

**Authors:** Mahmoud E. Khani, Zachery B. Harris, Omar B. Osman, Juin W. Zhou, Andrew Chen, Adam J. Singer, M. Hassan Arbab

**Affiliations:** 1 Department of Biomedical Engineering, Stony Brook University, Stony Brook, NY 11794 USA; 2 Department of Emergency Medicine, Stony Brook University, Stony Brook, NY 11794 USA

**Keywords:** Terahertz optics, Imaging and sensing, Optical spectroscopy, Biophotonics

## Abstract

We present an automatic classification strategy for early and accurate assessment of burn injuries using terahertz (THz) time-domain spectroscopic imaging. Burn injuries of different severity grades, representing superficial partial-thickness (SPT), deep partial-thickness (DPT), and full-thickness (FT) wounds, were created by a standardized porcine scald model. THz spectroscopic imaging was performed using our new fiber-coupled Portable HAndheld Spectral Reflection Scanner, incorporating a telecentric beam steering configuration and an f-$\theta$ scanning lens. ASynchronous Optical Sampling in a dual-fiber-laser THz spectrometer with 100 MHz repetition rate enabled high-speed spectroscopic measurements. Given twenty-four different samples composed of ten scald and ten contact burns and four healthy samples, supervised machine learning algorithms using THz-TDS spectra achieved areas under the receiver operating characteristic curves of 0.88, 0.93, and 0.93 when differentiating between SPT, DPT, and FT burns, respectively, as determined by independent histological assessments. These results show the potential utility of our new broadband THz PHASR Scanner for early and accurate triage of burn injuries.

## Introduction

In 2018, approximately 416,000 patients were treated for burn injuries in emergency departments in the United States^1^. The depth of injury in a cutaneous burn determines its potential for spontaneous healing or the need for surgical intervention, thus guiding the course of clinical management and the treatment plan. Burn injuries are designated into three categories: superficial (first-degree), partial-thickness (second-degree), and full-thickness (third-degree)^2^. The thermal insult in superficial burns is confined to the epidermis. Therefore, they will generally heal on their own through the re-epithelialization process^3^. In full-thickness burns the entire dermis layer, and often the underlying hypodermis, is necrotic, and therefore they require surgical intervention (skin grafting)^4,5^. Meanwhile, some of the partial-thickness burns heal spontaneously (i.e. superficial partial-thickness), whereas others will progress to a full-thickness state over the course of the natural inflammatory response to the burn (i.e. deep partial-thickness), which usually lasts a few days post-burn^6,7^. This intermediate group is largely responsible for inaccuracies in burn triage and therefore poor wound healing outcomes^8^. Early diagnosis and timely treatment, in the form of excision and grafting of deep burns, are therefore crucial in improving and expediting the healing process, reducing the infection rate, and avoiding hypertrophic burn scars^9–11^. Although the current clinical assessment technique, based on the visual examination of a burn injury, is effective in differentiation between superficial and full-thickness burns, classification of intermediate burns is largely subjective and remains the main source of error in burn triage^8^. The accuracy rate of clinical assessment for partial-thickness burns has remained as low as 60–75$\%$ several days post burn induction^12^, and significantly lower (about 50$\%$) for a diagnosis achieved within 24–48 h^6^. This is mainly due to the dynamic nature of the partial-thickness burns during the first 48–72 h after injury^13^.

The low accuracy in timely assessment of partial-thickness burns has motivated the development of a wide range of objective diagnostic modalities based on various imaging techniques. Different optical modalities, such as laser Doppler imaging^14^, indocyanine green (ICG) video angiography^15^, diffuse reflection near-infrared spectroscopy^16,17^, orthogonal polarization spectral imaging (OPSI)^18^, imaging by spatially-modulated structured light, also known as spatial frequency-domain imaging (SFDI)^19^, laser speckle imaging (LSI)^20^, photoacoustic microscopy (PAM)^21^, and polarization-sensitive optical coherence tomography (PSOCT)^22^, have been explored for that purpose. These techniques mostly focus on monitoring perfusion^15,23^, alterations in the cellular metabolism^24^, or the patency of the tissue microvasculature^18^. A major drawback of current optical techniques in the visible regime is their limited penetration depth, confining them to superficial blood flow. However, the analysis of deeper tissues would be more beneficial for differentiating the partial-thickness burns^6,25^. Additionally, most of these techniques have a small field of view, resulting in long acquisition times for larger wounds, which renders them more susceptible to the artifacts caused by the patient’s movements over time^20,26^. Among the aforementioned techniques, LDI offers the best estimates of burn injury severity^27^, identifying the full-thickness burns requiring surgical intervention with 95$\%$ accuracy^28^. However, LDI is less accurate when utilized within the first 24–48 h of injury^14,27^. Other wound healing studies indicated that it is only on day 3 post-burn that the severity assessment using LDI is significantly more accurate than the routine clinical assessment techniques^14^.

Terahertz time-domain spectroscopy (THz-TDS) has emerged as a noninvasive and objective tool for assessment of various biological tissue types, including determining corneal hydration^29–32^, diagnosis of brain tumors^33–35^, diabetic foot syndrome^36^, delineating breast cancer tumor margins^37–41^, and classification of burn injuries^42,43^. In burn diagnosis applications, it was shown that the reflectivity of the full-thickness burns in rodent models is higher compared to the normal skin over a frequency range between 0.2 and 1 THz^44^. This increased THz reflectivity was explained by the post-burn formation of the interstitial edema. Moreover, the double Debye dielectric relaxation model of healthy human skin^45,46^ was extended to model the THz response of the less severely burned tissue samples. More recently, it has been shown that THz waves are not only sensitive to the post-burn formation of interstitial edema, but also to the density of skin structures^47^. A linear combination of broadband THz reflectivity and spectral slope was correlated with the density of discrete scattering structures in the skin layers. Additionally, in an in vivo porcine pseudo-scald model, it was shown that a similar combination of THz hyperspectral parameters, i.e., the area under the reflectivity curve and the roll-off spectral slope, introduced as a novel THz *Z*-metric value, can differentiate between burn injuries with burn depth exceeding or below 350 $\mu$m, as determined by vimentin immunohistochemistry^48^. The *Z*-metric was further optimized to differentiate burns into two categories: 1. depth of injury greater than 50$\%$ of the dermis thickness, and 2. depth of injury less than 50$\%$ of the dermis thickness^49^. Moreover, it was reported that the longitudinal variation of the *Z*-metric in superficial partial-thickness burns is different from deep partial-thickness and full-thickness burns, and therefore it can be used for monitoring the wound healing outcomes.

Previous THz-TDS studies for burn assessment have utilized point-spectroscopy measurements, which are unable to capture the heterogeneity of the tissue by forming spectral images. In this work, we have addressed this limitation by demonstrating broadband THz spectroscopic imaging of graded burn severities in an in vivo porcine model. To enable large animal imaging without suffering from motion artifacts, all optical components were placed inside a fiber-coupled alignment-free handheld housing. A motorized gimbal mirror and a f-$\theta$ scanning lens were used in the handheld housing to raster scan the THz waves over a 27 $\times$ 27 mm$^2$ field-of-view. To examine the potential of THz-TDS for early diagnosis of partial-thickness burns, we compared the THz spectra measured 1 h after burn induction (Day 0) to histology measurements collected four days post-burn, when the dynamic burn wounds typically reach their maximum depth. Supervised machine learning algorithms using support vector machines, naive Bayes classifiers, discriminant analysis classifiers, and ensemble learning were developed and employed to classify the in vivo burn injuries into three groups, namely superficial partial-thickness (less than 40$\%$ dermal depth), deep partial-thickness (between 40 and 80 $\%$ dermal depth), and full-thickness (above 80 $\%$ dermal depth). The area under the curve (AUC) in the receiver operating characteristic (ROC) plots using support vector machines (SVM) with a Gaussian kernel reached 94$\%$ for classification of superficial partial-thickness burns, 89$\%$ for deep partial-thickness burns, and 91$\%$ for full-thickness burns. These results confirm that the THz-TDS measurements and machine learning techniques can be used together to yield accurate and early assessments in acute burn injuries.

## Methods

### THz time-domain spectroscopy

**Figure 1:**
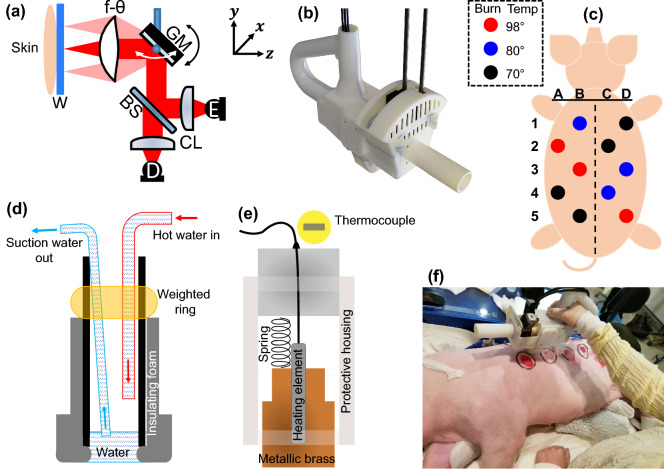
(**a**) The optical components inside the handheld housing (W: imaging window, GM: gimbaled mirror, BS: beam splitter, CL: collimating and refocusing lenses, E: emitter PCA, D: detector PCA), the black and white arrows show the mirror movement in *y* and *x* directions, respectively, (**b**) the 3D-printed handheld housing (dimensions: 37.3 $\times$ 14 $\times$ 25.1 cm$^3$), (**c**) the pattern of the distribution of the burn temperature on the dorsal side of the subject, (**d**–**e**) the side view schematics of the scald and contact devices designed for burn induction, respectively, (**f**) demonstration of in vivo THz spectroscopic imaging of burn injuries using the PHASR Scanner.

THz-TDS measurements were obtained using our PHASR (Portable HAndheld Spectral Reflection) Scanner, described previously^50^. Briefly, we incorporated the TERA ASOPS dual-fiber-laser spectrometer (Menlo Systems, Inc., Newton, NJ, USA) in our PHASR Scanner, where THz pulses are generated by optical excitation of a photoconductive antenna (PCA) using 1560 nm pulses of a femtosecond laser at 100 MHz repetition rate. A second synchronized femtosecond laser at a close repetition rate with a fixed $\Delta f =$ 100 Hz offset generates the probe pulses. This offset rate allows for high-speed scanning over ten nanoseconds of time delay between pump and probe pulses without a mechanical delay line. The optical components of the spectroscopic imaging setup, schematically shown in Fig. 1a, were placed inside the handheld housing of the PHASR Scanner, shown in Fig. 1b. Broadband THz beams generated by the emitter PCA are collimated using a TPX lens (Menlo Systems, Inc., Newton, NJ, USA) with 50 mm focal length. A high-resistivity silicon beam splitter guides the collimated beams toward a mirror mounted on a 2-axis motorized heliostat system, composed of a goniometer and a rotational stage, which raster scans the beam over a custom-made f-$\theta$ lens^51^. In a telecentric f-$\theta$ scanning system, collimated beams passing through the front focus of an f-$\theta$ lens

at a deflection angle $\theta$ are focused to a true planar surface at a distance $f\times \theta$ from the optic axis^52^. Therefore, the focused beam remains parallel to the optic axis and has a constant spot-size at the target plane^52^. In this study, we used a high-density polyethylene (HDPE) f-$\theta$ lens with a 40 mm focal length. Upon reflection from the sample, THz beams retrace the path of the incident beams back to the beam splitter, where they are directed towards a focusing TPX50 lens and collected by the detector PCA. Using this THz PHASR Scanner design, an area of approximately 7.29 cm$^2$ (27 $\times$ 27 mm$^2$) was scanned in about three minutes, while recording the full THz-TDS spectrum of each 1 mm$^2$ pixel area. Table 1 provides the performance metrics of the PHASR Scanner.

**Table 1:** THz scanner performance parameters.

Parameter	Value
Housing dimensions	37.3 $\times$ 14 $\times$ 25.1 cm$^3$
Focus size	3.1 $\times$ 2.4 mm$^2$
Focus depth	7.25 mm
Field of view	27 $\times$ 27 mm$^2$
Pixel size	1 $\times$ 1 mm$^2$
Number of time averages	20 averages per pixel
Scan time	3 min per field of view

A custom MATLAB (Mathworks Inc., Natick, MA, USA) software was used for controlling the hardware and acquiring the THz-TDS measurements.

### Burn protocol

We conducted an acute in vivo scald study in a porcine model. The experimental protocol used in this study was approved by the Institutional Animal Care and Use Committee (IACUC) at the Stony Brook University. All methods reported here were performed in accordance with the relevant guidelines and regulations of the IACUC and follows the recommendations in the ARRIVE guidelines^53^. The animal model used was female Yorkshire pigs, weighing 45–50 kg. Prior to inducing the burns on the first day of the study (Day 0), the animal was sedated through an intramuscular (IM) injection of a pre-anesthetic cocktail consisting of ketamine (20 mg/kg), xylazine (2.2 mg/kg), acepromazine (0.1 mg/kg), and atropine (0.02 mg/kg), and then anesthetized with a continuous flow of 0.5–5$\%$ isoflurane. The animal was kept on isoflurane throughout the imaging process and monitored continuously. On Day 0, multiple scald or contact burns were created on the dorsal side of the subject^54^, following the pattern shown in Fig. 1c. The scald device, which is shown schematically in Fig. 1d, was composed of a foam-wrapped steel pipe, an inflow hot water tube, and an outflow suction tube. When water in a heating reservoir reached the desired temperature using an immersion heating circulator, it was pumped into the steel pipe placed on the pig skin. After being in contact with the skin for a specific duration, e.g., ten seconds in this study, the hot water was removed using a vacuum pump (Adafruit Industries, New York, NY, USA), attached to a vacuum flask. The contact device, shown schematically in Fig. 1e, was composed of a metallic brass bar, a heating element, a thermocouple, and a spring loaded tube to maintain constant pressure (2 kg/6.25 cm). Burn severity grades were created at multiple sites by exposure to hot water or brass bar at 70 $^\circ$C, 80 $^\circ$C, and 98 $^\circ$C, shown in Fig. 1c by black, blue, and red, respectively. To control for the anatomical and skin thickness variations, the locations of each burn induction temperature group were uniformly varied and distributed on the dorsal side of the animal subject. Moreover, the locations of the burns were chosen such that they were 4 cm apart in all horizontal and vertical directions. This distance is deemed sufficient to avoid the effect of inflammatory response of each burn on its adjacent burns^55^. Following the burn induction, buprenorphine (0.005-0.02 mg/kg) was administered IM and a 72-h slow-release transdermal fentanyl patch (50 $\mu$g/g) was placed proximal to the tail. The THz-TDS measurements on Day 0 were obtained after the burns were debrided by gently scraping with the blunt end of forceps, consistent with the wound debridement step in routine clinical burn care. After obtaining the THz-TDS measurements using the PHASR Scanner, as is shown in Fig. 1f, triple antibiotic ointment was applied to each burn wound to prevent infection. The burns were then bandaged by covering the wounds with a transparent Tegaderm sheet (3M, Saint Paul, MN, USA). Finally, the midsection of the pig was wrapped with flexible gauze bandage and adhesive Tensoplast (BSN Medical, Hamburg, Germany). On Day 4 of the study, 96 h post burn induction and prior to imaging, the animal was sedated and anesthetized with the same procedure used on Day 0.

### Histopathology

**Figure 2:**
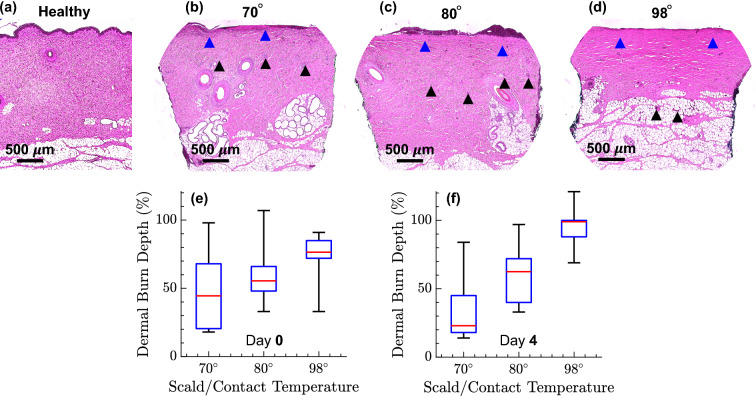
(**a**–**d**) Microscopic images ($5\times$ magnification) of the H&E-stained biopsies from the healthy skin (control experiment) and three representative burns created using 70$^{\circ }$, 80$^{\circ }$, and 98$^{\circ }$ scald, respectively, the blue and black arrows indicate the areas of collagen denaturation and vascular occlusion, respectively, (**e**) the dermal burn percentage measured on the biopsies on Day 0, and (**f**) Day 4.

Four-millimeter punch biopsies were collected on Days 0 and 4 of the study (corresponding to the peak of the inflammatory response^56^) for histological assessment. The excised biopsies were fixed in 10$\%$ buffered formalin for 24 h and then paraffin embedded. They were then stained with hematoxylin and eosin (H&E). Microscopic images of the H&E-stained biopsies for healthy skin and representative burn wounds are shown in Fig. 2a–d. The burn depth was assessed by measuring the deepest point of injury, which can be characterized by microvascular occlusion, collagen discoloration, or necrosis of follicular, mesenchymal, and adipocyte cells^54^, as identified in Fig. 2b–d. Blind evaluation was performed by a board-certified histopathologist. The depth of injury was normalized by the total dermal thickness and presented in percentage of dermis thickness. Figure 2e, f show the dermal burn depth percentage on Days 0 and 4, respectively, using box plots, where the red lines show the group medians and each box represents the 25$\%$ to 75$\%$ of the burn depth range in each temperature group, and the whiskers covering the full range of the obtained data. It can be noted that progressively deeper burns are observed with increasing scald temperature on Day 4 post-burn, when the peak inflammatory response of the tissue to thermal damage occurs and the burns reach their maximum depth^56^. Accordingly, tissues with less than 40$\%$ burn depth in the dermis layer were grouped as the superficial partial-thickness (SPT) category. Tissues with burn depth between 40$\%$ and 80$\%$ of dermal thickness formed the deep partial-thickness (DPT) group, and samples having larger than 80$\%$ dermal burn depth, including those with a damaged hypodermis, were grouped as the full-thickness (FT) category. It should be noted that our burn induction devices, which are designed and fabricated following the techniques demonstrated in^57,58^, result in highly consistent and fairly homogeneous burns over the same location. In addition, the 40$\%$ burn thickness range encompassed by each burn severity group is large enough to account for slight variations in the burn thickness over the same burn location.

### Signal processing

**Figure 3:**
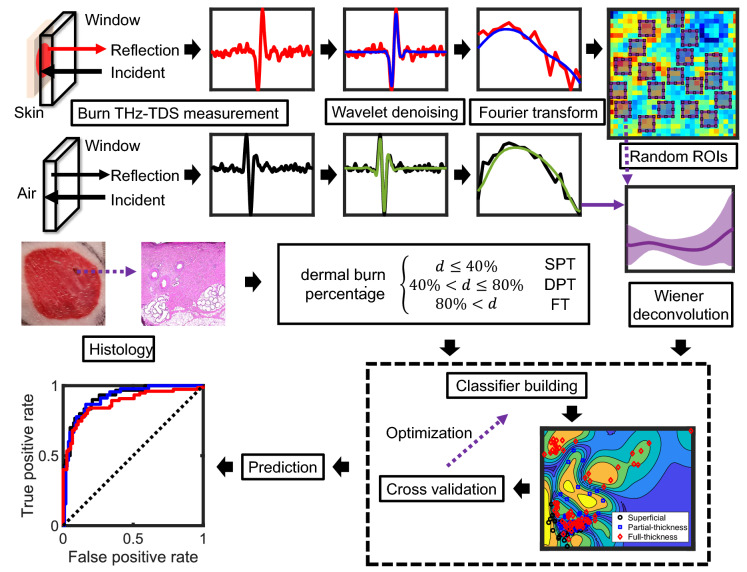
The workflow of training and testing the classifiers for diagnosing a burn injury’s severity into one of the SPT, DPT, and FT groups. After wavelet denoising and Wiener deconvolution of the measured THz pulses, an image was formed using the bandwidth-limited area under the reflection spectral amplitude curve. Fifteen 4 $\times$ 4-pixel regions of interest (ROI) were selected randomly over each image. The burn severity labels were assigned using the dermal burn percentage (*d*) in biopsies collected on Day 4 (SPT ($d<40\%$), DPT ($40\%<d<80\%$), and FT ($80\%>d$)). Each classifier’s specific hyper-parameters were optimized using Bayesian optimization during training. Additionally, to avoid over-fitting, each classifier was fivefold cross validated. The performance of various machine learning techniques (i.e., SVM, NB, LDA, and boosted LDA) was evaluated based on the area under the receiver operating characteristic (ROC) curves.

Signal processing in both time and Fourier domains was important in extracting relevant spectroscopic information for burn assessment. In in vivo experiments, because of the relatively small number of possible averages (twenty averages per pixel in this case), the noise contribution before and after the main THz pulse was noticeable. Because the acquisition time is directly proportional to the number of time averages, acquiring many averages at each pixel is time-consuming and can result in mechanical and motion artifacts. Therefore, we implemented two data-driven signal processing steps to achieve a higher signal to noise ratio (SNR) over a larger bandwidth even with the fewer number of time averages. First, the THz waveforms were denoised using wavelet shrinkage^59,60^. The wavelet denoising algorithm was designed based on the maximal overlap discrete wavelet transform of the time-domain THz signals and the decomposition level-dependent wavelet thresholding^61^. Accordingly, at each decomposition level, one threshold was set based on two time windows in the signal, i.e., before and after the main THz pulse, and signals were modified using wavelet hard thresholding^59,62^. In the second pre-processing step, the THz pulse reflected from the imaging window-skin interface was deconvolved using a reference measurement obtained from the window-air interface. Here, we implemented a Wiener deconvolution scheme, where the SNR at each frequency point is accounted for to prevent large spectral value artifacts caused by the ill-posed deconvolution implementation^59,63^. A summary of the signal processing steps and example spectral data are shown as part of the flowchart in Fig. 3.

### Machine learning classifier

Figure 3 exhibits the machine learning workflow for THz spectral analysis. The first two steps in the signal processing flowchart, i.e., wavelet denoising and Wiener deconvolution, were described in the earlier section. THz images are formed using the bandwidth-limited area under the reflection spectral amplitude curves. To create the predictor space, fifteen 4 $\times$ 4-pixel regions of interest (ROI) were selected randomly over each of the ten burns, where each pixel represented the THz spectral response of a 1 mm$^2$ burned tissue. It has been shown that the Mie scattering by granular particulate structures gives rise to significant spectral artifacts in the THz regime^64^, which can be mitigated using signal processing^65,66^ or ensemble averaging^67^ techniques. To account for the scattering effects caused by the heterogeneous skin appendages, the average THz reflectivity of the sixteen pixels in each ROI was used. In other words, the deconvolved spectrum averaged over pixels in each ROI in the frequency range between 0.1 and 0.5 THz, was used as a single observation in later classification stages. Additionally, the random selection of the ROIs, in contrast to a deterministic selection, and repeating the random trials for ten iterations, avoid the classifiers’ performance to be biased toward a specific configuration of the ROIs. Burn severity classification labels were assigned using the dermal burn percentage in biopsies collected on Day 4.

Different machine learning algorithms, including support vector machine (SVM), naive Bayes (NB) classifier, and linear discriminant analysis (LDA) classifier, were modeled separately to predict the severity designation of a burn tissue based on its THz spectral response. The SVM algorithm has been used for classification of THz spectra of various pathological tissues, such as breast cancer^39,68^, colon cancer^69^, and gastric cancer^70^. The SVM classifier utilizes an iterative approach to maximize the boundary between different classes, while using the minimal amount of the support vectors^71^. In this study, we compare the performance of SVM classifiers with linear, polynomial (n$=2, \,3,\,{\text { and }}\,4$), and Gaussian kernel functions^72^. The NB classifier leverages the Bayes theorem, with the two assumptions that predictors within each class are independent, and predictors have similar effects on the outcome^73^. The NB classifier estimates the prior density function of the predictors within each class, and models the posterior probabilities following the Bayes theorem^74^. Accordingly, an observation is assigned to the class that yields the maximum posterior probability. The LDA classifier assumes predictors within each class form a Gaussian mixture distribution^74,75^. In LDA, the covariance matrix of the Gaussian mixture models in different classes remains the same, while their means vary. The classifier assigns an observation to the class that minimizes an expected classification cost^74^. Finally, we examine Ensemble learning techniques, combination of multiple learning algorithms to improve the classification performance^74^, based on the boosted discriminant classifiers^76^. In the adaptive boosting (AdaBoost) of multiple classifiers, an equal weight is given to all the training instances initially. However, subsequent learners are tweaked to favor those instances misclassified by the previous classifiers^76,77^.

In experiments with limited sample size, cross-validation is used to avoid over-fitting and to estimate how accurately a predictive model will perform on an independent set not included in training^74^. In this work, we also used fivefold cross validation to avoid over-fitting and to evaluate the predictive ability of our classification models. In fivefold cross validation, each classifier hyper-parameters are set based on only 80$\%$ of the samples, while 20$\%$ of the samples are reserved for calculating the test error rate. Furthermore, by iterating this process for 5 times, while completely different samples are used as the test set of each iteration, we avoid the dependence of classifiers’ performance metrics on how the samples are divided into the train and test sets. Each model’s specific hyper-parameters were optimized over the training set using Bayesian optimization^78^. In the results section, we compare the performance of various machine learning techniques based on the area under the receiver operating characteristic (ROC) curves, i.e., the true positive rate (TPR) versus the false positive rate (FPR), obtained for differentiation between the three burn severity groups.

## Results

**Figure 4:**
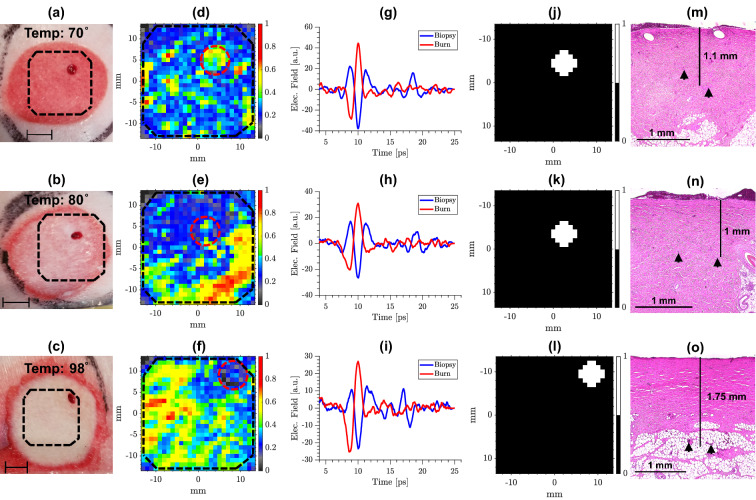
(**a**–**c**) Digital images of the burns created using the scald device at 70$^{\circ }$, 80$^{\circ }$, and 98$^{\circ }$, respectively, the scale bars are 10 mm, and the dashed black lines show the field-of-view of the scanner, (**d**–**f**) THz images of the burns in (**a**–**c**), respectively, formed using the area under the reflected spectral amplitude curves between 0.1 and 0.5 THz, the dashed red circles outline the biopsy locations, (**g**–**i**) representative THz-TDS pulses measured from the burn (solid red line) and the biopsy (solid blue line) locations at each burn, (**j**–**l**) determination of the pixels associated with the punch biopsies identified using the phase of the Fabry–Perot reflections in the THz-TDS pulses, (**m**–**o**) microscopic images ($5\times$ magnification) of the H&E-stained biopsies of the burns shown in (**a**–**c**), respectively, the vertical solid black lines show the average depth of the deepest point of injury (black triangles).

Figure 4 compares digital images obtained from the burns on Day 0 with THz images on Day 0 formed using the area under the reflected spectral amplitude curves between 0.1 and 0.5 THz at each pixel. Images in Fig. 4a–c illustrate representative burns created using the scald device at 70$^{\circ }$, 80$^{\circ }$, and 98$^{\circ }$, respectively. The length of the scale bars in Fig. 4a–c is 10 mm. The dashed black lines show the scanner’s field-of-view on each burn, carefully placed so that the four-millimeter punch biopsies collected earlier were identified in the upper right quadrant of each image. Figure 4d–f show the corresponding THz images of the burns in Fig. 4a–c, respectively. The dashed black lines in Fig. 4d–f also delineate the same field-of-view in Fig. 4a–c. Additionally, the dashed red circles outline the location of biopsies in each image. Figure 4g–i show the representative THz-TDS pulses measured from the burn (solid red line) and the biopsy (solid blue line) locations in Fig. 4d–f, respectively. The biopsy locations. which serve as fiducial markers for imaging coregistration, can be identified using the phase of the Fabry–Perot internal reflections in the imaging window using the time-domain THz pulses, as illustrated in Fig. 4g–i. Briefly, the air gaps formed between the imaging window and the tissue at the biopsy locations result in the phase change in Fabry–Perot reflections, which are utilized to delineate the pixels associated with the biopsy locations, as shown in Fig. 4j–l.

Figure 4j–l show binary images corresponding to Fig. 4d–f, where pixels identified as biopsy are equal to one, whereas pixels elsewhere equal to zero. The biopsy pixels are excluded from the classification algorithm, since the burned tissue in those pixels is removed. Figure 4m–o show the microscopic images (5 $\times$ magnification) of the H&E-stained biopsies from the burns in Fig. 4a–c, respectively. The dermal burn depth, which is determined based on the deepest point with microvascular occlusion (shown using the black arrows), is also reported for each burn in Fig. 4m–o. It can be noted that despite the lower scald temperature, the 70$^{\circ }$ burn in Fig. 4a has resulted in a 0.1 mm-deeper dermal burn in comparison to the 80$^{\circ }$ scald burn shown in Fig. 4b. This discrepancy between the induction temperature, shape, and depth of the burn wounds results from the complex heterogeneity of the skin and its constituents, and therefore, it necessitates the use of non-invasive and objective modalities that enable a deeper monitoring of the skin condition, such as the THz time-domain spectroscopy.

**Figure 5:**
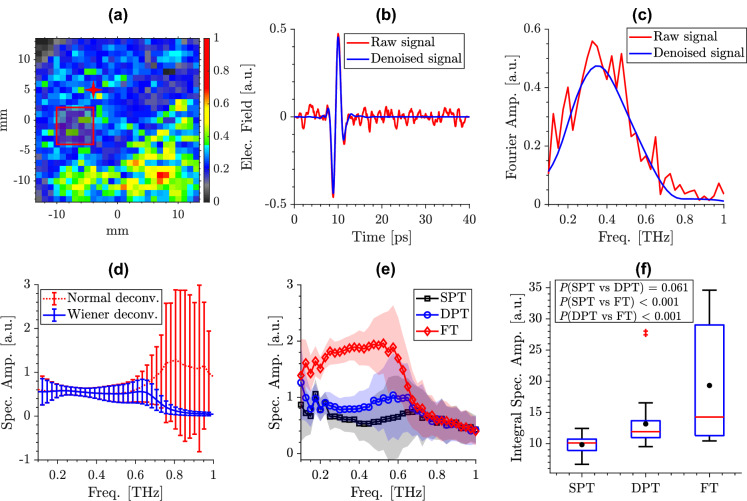
(**a**) A representative THz image of a burn created with 70 $^\circ$C scald device over a 27 by 27 mm$^2$ field-of-view. The color axis shows the normalized area under the reflection spectral amplitude curves between 0.1 and 0.5 THz. (**b**) the wavelet denoising outcome in an example THz-TDS pulse selected from a pixel marked by the red star in (**a**), and (**c**) the Fourier amplitude of the signals shown in (**b**), (**d**) comparison between normal and Wiener deconvolution in a 4 $\times$ 4-pixel ROI shown using the red box in (**a**), where Wiener deconvolution effectively avoids the high-frequency spikes originated from an ill-posed deconvolution implementation, (**e**) the average spectral amplitude of reflectivity obtained after the Wiener deconvolution, along the standard deviation over the pixels of three ROIs, each selected randomly from a different burn grade example, i.e., SPT (black line, square markers), DPT (blue line, circle markers), FT (red line, diamond markers), (f) box plots comparing the area under the spectral amplitude curves in all burns (composed of thirty 4 $\times$ 4-pixel ROIs randomly selected from the burns in each group) over 0.1–0.5 THz, using a one-way ANOVA, the difference between SPT and FT groups (${p}$ < .001), and DPT and FT groups (${p}$ < .001) is statistically significant, while the ${p}$(SPT vs DPT) = 0.061.

Figure 5 shows representative results in different steps of the signal processing flowchart explained earlier. Figure 5a shows a representative THz image of a SPT burn created using 70 $^{\circ }$ scald device. The field of view shown by the x and y axes are 27 by 27 mm, and the color axis represents the normalized area under the reflection spectral amplitude curves between 0.1 and 0.5 THz. Here, the burn depth was determined to be 22$\%$ of the dermis thickness. Figure 5b, c shows the wavelet denoising outcome in an example THz-TDS pulse selected from a pixel marked by the red star in Fig. 5a. It can be noted that although the noise surrounding the main THz pulse is prominent in the raw signal (red trace) in Fig. 5b, wavelet shrinkage has effectively removed all such contributions in the denoised signal (blue trace), while the main pulse remains nearly intact.

Moreover, it can be observed in Fig. 5c that while spectral fluctuations abound in the Fourier transform of the raw THz pulse, they are removed in the wavelet shrinkage outcome. Such spectral artifacts can diminish the accuracy in automatic classification, and their removal enhances the precision in differentiation between the burn grades. Figure 5d compares the conventional and Wiener implementations of deconvolution in a 4 $\times$ 4-pixel ROI selected in the burn, as shown in Fig. 5a with the red box. It can be noted that the Wiener implementation effectively avoids the higher-frequency spikes after 0.6 THz, which are not diagnostic of the burn tissue and only appear due to the ill-posed deconvolution^59^. Figure 5e compares the Wiener-deconvolved spectral amplitude of reflectivity for three ROIs, each selected randomly from a different burn grade example, i.e., SPT, DPT, and FT burn groups. Each line in Fig. 5e illustrates the average spectral amplitude over the pixels of an ROI plotted with the standard deviation as the error bars. It can be noted that the spectral amplitude of FT burns is higher than the spectral amplitude of DPT and SPT burns. The higher spectral amplitude in deeper burns is mainly associated with the formation of the interstitial edema, which results in a change in the skin’s refractive index, yielding larger reflections from the imaging window-skin interface. Figure 5f compares the area under the spectral amplitude curves within 0.1–0.5 THz for the three burn designations in all ten burns (composed of thirty 4 $\times$ 4-pixel ROIs randomly selected from the burns in each group) using box plots. Here, the black circles and the red lines indicate the mean and median of each group, respectively. The bottom and top edges of each box indicate the 25th and 75th percentiles in each group, and the whiskers extend to the data points within the 1.5 times the interquartile range from either edge of the box. The outliers outside this range are plotted individually using the red ‘+’ symbol. A one-way analysis of variance (ANOVA) at $\alpha =0.05$ level of significance confirms that the difference between SPT and FT groups (${p}$ < .001), and also the difference between DPT and FT groups (${p}$ < .001) is statistically significant, while the difference between SPT and DPT was not significant (${p}$ = 0.061). Comparing the area under the spectral amplitude curves in Fig. 5f with the dermal burn percentage reported in Fig. 2f suggests that THz spectral amplitudes measured immediately post-burn can potentially predict the eventual burn depth on Day 4, which typically increases in response to the peak inflammatory processes of the tissue to a thermal insult and occurs 72–96 h post burn induction^56^. It should be noted that results in Fig. 5f confirm the need for using full spectroscopic information in conjunction with supervised machine learning algorithms to improve the accuracy of burn classification using THz-TDS measurements. In the final section of this paper, we will explore such a possibility using supervised machine learning techniques.

**Figure 6:**
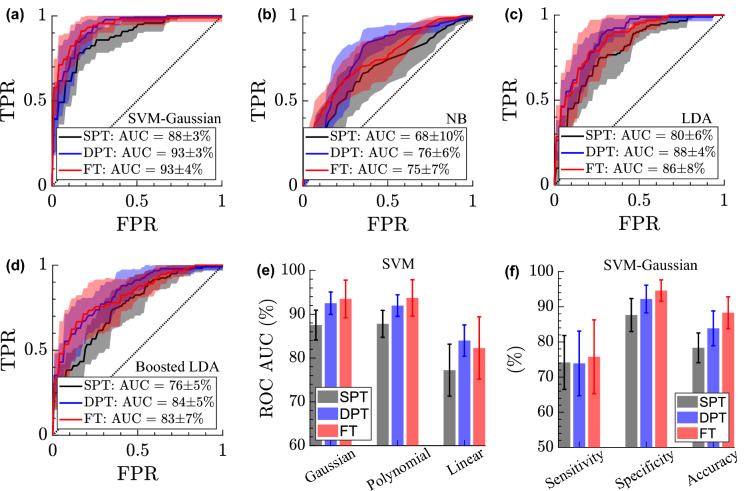
(**a**–**d**) The ROC curves obtained using the Gaussian Support Vector Machine (SVM), Naive Bayes (NB), Linear Discriminant Analysis (LDA), and AdaBoosted LDA classifiers for identification of SPT (black lines), DPT (blue lines), and FT burns (red lines), (**e**) comparison of the area under the ROC curves obtained using the SVM classifiers with Gaussian, polynomial (quadratic, cubic, and the fourth power), and linear kernel functions, (**f**) the sensitivity, specificity, and accuracy rates in classification of each burn group obtained using the Gaussian SVM, the error bars in the ROC curves and the bar plots show the standard deviation over the 10 iterations of each classifier with different random ROIs selected during each iteration.

Figure 6a–d show the ROC curves, by plotting the True Positive Rate (TPR) versus the False Positive Rate (FPR) as the discrimination threshold on a classifier’s scores is varied. The performance of the classifiers in identification of SPT (black lines), DPT (blue lines), and FT burns (red lines), i.e., differentiation of each group from the other two groups, or one versus all, are plotted separately. In the methods section, we explained that to account for tissue heterogeneity, fifteen 4 $\times$ 4-pixel ROIs were selected randomly over each burn, as shown in Fig. 3, and the average spectral amplitude in each ROI was utilized as a single observation for building the classifiers. Here, each ROC curve shows the average and standard deviation over ten iterations of each classifier, with different ROIs randomly selected in each run.

The dotted diagonal lines show the ROC curve of a random classifier with no discrimination power. An ROC curve with a higher area under the curve (AUC) indicates a classifier with a higher accuracy rate, with an ideal classifier having an AUC of 100$\%$. Therefore, the AUC value can be used to evaluate and compare the performance of different classifiers. In Fig. 6a–d, the average and the standard deviation of AUC values of each classifier over the ten iterations are given in the legends, where different training and test sets have been utilized in each trial. Figure 6a demonstrates the ROC curves of the SVM classifier with a Gaussian kernel function, where the AUC in the classification of SPT, DPT, and FT burns is 88 ± 3$\%$, 93 ± 3$\%$, and 93 ± 4$\%$, respectively. Figure 6b–d shows the ROC curves obtained using the NB, LDA, and AdaBoosted LDA classifiers, respectively. It can be noted that the NB classifier has the lowest overall AUC values as compared to the other algorithms. Additionally, comparing the LDA and boosted LDA curves in Fig. 6c–d reveals that does not improve the AUC for classification of either of the three groups. Comparison between the SVM classifier in Fig. 6a with the other classifiers in Fig. 6b–d, considering the range of variation in the AUC values given as the standard deviation in the legends, reveals that the Gaussian SVM outperforms other classification models. To assess the effect of the kernel function in an SVM classifier model, Fig. 6e compares the AUC values for SVM classifiers with Gaussian, polynomial, and linear kernel functions. Here, the error bars show the standard deviation of AUC over the ten iteration tests. The AUC in classification of the SPT, DPT, and FT burns using the SVM classifier with a Gaussian kernel function is 88 ± 3$\%$, 93 ± 3$\%$, and 93 ± 4$\%$, respectively. These values for the SVM with a polynomial kernel function are 88 ± 3$\%$, 92 ± 2$\%$, and 94 ± 3$\%$, and for the SVM with a linear kernel function are 77 ± 6$\%$, 84 ± 4$\%$, and 82 ± 7$\%$. It should be noted that the power of the polynomial kernel function was set as an optimization parameter during the cross-validation. Comparing different SVM kernels based on the average and standard deviation in AUC, the performance of Gaussian and Polynomial kernels is identical, whereas the performance of the SVM classifier with a linear kernel function is inferior, signifying the importance of selecting an appropriate kernel function when using this algorithm. Finally, Fig. 6f shows the sensitivity, specificity, and accuracy in classification of each burn severity group using the SVM classifier with a Gaussian kernel function. The average sensitivity rates in differentiating SPT, DPT, and FT burns were determined to be 74$\%$, 74$\%$, and 76$\%$, respectively. The average specificity rates for classifying SPT, DPT and FT burns were found to be 88$\%$, 92$\%$, and 95$\%$, respectively, while the overall average accuracy rates were 78$\%$, 84$\%$, and 88$\%$. The high specificity rate achieved using our technique means that there are fewer false positive results in each group, which is highly desirable when the diagnostic test may be used to recommend invasive or costly procedures.

## Discussion

Our study shows the potential of the THz-TDS imaging for early and accurate assessment of burn injuries. We used a handheld THz time-domain scanner to classify burn injuries in an acute in vivo study of porcine scald burns. Utilization of our new PHASR Scanner based on THz asynchronous optical sampling (ASOPS) and a telecentric beam scanning system in a 3D-printed housing enabled alignment-free and high-speed broadband spectroscopic measurements inside an operating room environment. We also showed that by implementing appropriate signal pre-processing techniques, including wavelet denoising and Wiener deconvolution, common machine learning algorithms are able to classify different burn grades using THz spectroscopic features collected 1-h post burn induction. In particular, the support vector machine classifier with a Gaussian kernel function achieved above 89$\%$ area under the ROC curve for differentiation of SPT, DPT, and FT burns using the THz spectra collected only 1 h post burn induction. Importantly, the depth of burn injuries was determined by an independent and blinded histopathologist using biopsies obtained 4 days post burn induction, corresponding to the peak of the inflammatory response of the tissue. Because in routine burn care physicians often delay surgical interventions for a few days until the true depth of each burn can be discerned, the ability to predict the burn depth using THz spectra on Day 0 will allow for early and accurate determination of the treatment plan, which in turn can result in improved wound healing outcomes and infection prevention^5,9^.

One limitation of this study was the total number of observations available for training and testing the machine learning algorithms. Although cross-validation techniques and randomized iterative runs are well established counter-measures to potential overfitting in data scarce scenarios, it can impact quantification of sensitivity and specificity. Another limitation of studies performed with standardized burn induction protocols is that the robustness of the classification technique to various types of burn etiologies, such as burns caused by chemical or electrical energy, are not tested. Future work includes expanding experimental data sets, utilizing more advanced learning techniques, such as deep neural networks, and enhanced PHASR Scanner designs to improve the classification performance in differentiating burn injuries with various etiologies.

## Data Availability

The datasets used and analyzed during the current study are available from the corresponding author on reasonable request.
